# The cell cycle stage of bovine zygotes electroporated with CRISPR/Cas9-RNP affects frequency of Loss-of-heterozygosity editing events

**DOI:** 10.1038/s41598-022-14699-5

**Published:** 2022-06-24

**Authors:** Dennis Miskel, Mikhael Poirier, Luisa Beunink, Franca Rings, Eva Held, Ernst Tholen, Dawit Tesfaye, Karl Schellander, Dessie Salilew-Wondim, Carina Blaschka, Christine Große-Brinkhaus, Bertram Brenig, Michael Hoelker

**Affiliations:** 1 Institute of Animal Sciences, Animal Breeding, University of Bonn, Endenicher Allee 15, 53115 Bonn, Germany; 2 Department of Animal Science, Biotechnology and Reproduction of Farm Animals, Georg August University Goettingen, Burckhardtweg 2, 37077 Goettingen, Germany; 3 Department of Molecular Biology of Livestock, Institute of Veterinary Medicine, Georg August University Goettingen, Burckhardtweg 2, 37077 Goettingen, Germany; 4 Department of Biomedical Sciences, Animal Reproduction and Biotechnology Laboratory, Colorado State University, 3105 Rampart Rd, Fort Collins, CO 80521 USA

**Keywords:** Biotechnology, Animal biotechnology

## Abstract

At the embryonic level, CRISPR technologies have been used to edit genomes reliably and efficiently in various mammalian models, with Ribonucleoprotein (RNP) electroporation potentially representing a superior delivery method into mammalian zygotes. However, detailed insights of the interactions between varying technical settings as well as the time point of electroporation in a bovine zygote’s cell cycle on developmental metrics and the frequency and type of editing events are largely unknown. The present study uncovers that increasing pulse lengths result in higher Full Edit rates, with Mosaicism in Full-Edit embryos being significantly affected by adjusting RNP-electroporation relative to zygote cell cycle. A considerable proportion of Full Edit embryos demonstrated loss-of-heterozygosity after RNP-electroporation prior to S-phase. Some of these loss-of-heterozygosity events are a consequence of chromosomal disruptions along large sections of the target chromosomes making it necessary to check for their presence prior use of this technique in animal breeding. One out of 2 of these loss-of-heterozygosity events, however, was not associated with loss of an entire chromosome or chromosomal sections. Whether analysed loss-of-heterozygosity in these cases, however, was a false negative result due to loss of PCR primer sequences after INDEL formation at the target side or indeed due to interhomolog recombination needs to be clarified in follow up studies since the latter would for sure offer attractive options for future breeding schedules.

## Introduction

Clustered regularly interspaced short palindromic repeats (CRISPR) and CRISPR associated protein 9 (Cas9) are RNA guided endonucleases that have been shown to reliably cleave mammalian genomes^1^. By appropriating cellular DNA repair pathways, researchers have efficiently knocked out genes via non-homologous end-joining (NHEJ)^2^ or generated precise edits via homology directed repair (HDR)^3^, both providing highly attractive options for future animal breeding programs. Progress in traditional bovine breeding programs, however, is often made difficult by high genetic correlations between desired and undesired traits. The capacity to precisely edit certain traits by taking advantage of these new “breeding methods” could greatly increase the speed and precision of breeding programs by decoupling these outcomes. Additionally, gene defects are often the result of single mutations. Again, this gives researchers the potential to permanently repair mutated genes or to introduce desired traits to individuals or to the bovine gene pool via germline editing. While somatic cell nuclear transfer is a method that has been shown to work with edited donor cells^4^, the inherent inefficiency of the cloning process itself^5^, as well as potential epigenetic deregulation^6^ and concerns about cloning in general in western countries, suggest that work in fertilized zygotes may offer a more desirable option for gene editing bovine embryos and subsequent offspring.

At the embryonic level, CRISPR technologies have been used to reliably and efficiently edit genomes in various mammalian models using microinjection^7–9^ of guide RNA and Cas9 expression plasmids^10^, guide RNA and Cas9 mRNA^11^ as well as RNPs.

Recent studies conducted in the mouse have also demonstrated that the efficiency of electroporation of guide RNA and Cas9 mRNA but especially of RNPs may make it a superior delivery method into mammalian zygotes^12–16^. These studies suggest significantly higher survival rates and efficiencies of electroporation over microinjection in rats, mice, and pigs. Moreover, due to the technical expertise, micromanipulation equipment, and time required to perform microinjection of individual zygotes, the relative ease and high throughput of grouped electroporation is a promising alternative. While one initial study has shown the general possibility of generating gene knockouts in bovine embryos via electroporation with Cas9^17,18^, detailed investigations of the interactions between contrasting technical settings (e.g. field strength and pulse duration) as well as biological parameters (timing electroporation relative to zygote’s cell cycle state) on subsequent survival, cleavage, developmental and editing rates are missing. Although of high importance for bovine breeding, a detailed analysis of the effect of technical settings in the context of RNP-electroporation on developmental rates and gene editing events produced has also not been reported, nor have the effects on mosaicism and repair mode. In a previous study it was suggested that earlier introduction of Cas9 mRNA by injection after onset of fertilization reduced the rates of mosaicism in developed bovine embryos compared to later introductions^19,20^. While this could be explained by the fact that mRNA must be translated into protein, requiring time before Cas9 activity can be expected, no study has analysed the effect of the time of CRISPR-RNP electroporation relative to the zygote’s cell cycle stage on editing events and rates of mosaicism in bovine embryos. Keeping in mind that studies have demonstrated that the S-phase of the zygote cell cycle starts as early as 10 h after fertilization and is completed around 18 h after fertilization in bovine^21^, we hypothesised that editing efficiency and editing type might depend on temporal orchestration of electroporation relative to the S-phase in fertilized bovine zygotes.

Therefore, the aim of the present study was first to unravel the effects of contrasting technical settings (pulse duration) on developmental rates as well as editing efficiencies after electroporation of bovine zygotes with CRISPR RNP. Secondly, we tested the effect of the time of CRISPR-RNP electroporation relative to the cell cycle stage of bovine zygotes on developmental metrics and on the type and frequency of genome editing events, with special attention on mosaicism, full edits, biallelic editing as well as homozygous biallelic editing. Likewise, new scientific strategies were employed to analyse Loss-of-heterozygosity editing events and potential breakage of chromosomes harbouring the target site.

## Results

### Embryo developmental metrics

Bovine in vitro derived IVF zygotes were allocated into two treatment groups and were electroporated 10 and 18 h post insemination (HPI), using 25 V (voltage) for 5 pulses lasting 1, 2 or 3 ms (pulse length) (2 × 3 factorial model). The highest blastocyst rates were observed after 18 HPI and 1 ms pulses (38.7 ± 5.9%). The lowest rates were observed after 10 HPI and 3 ms pulses (21.2 ± 3.6%). The blastocyst rates per electroporated zygote were significantly affected by field strength (p < 0.05, Table 2) with blastocyst rates increased with shorter PL both for embryos electroporated at 10 h or 18 hpi (Table 1). Conversely, time of electroporation did not affect developmental rate to the blastocyst stage.

**Table 1:** Effect of time post insemination and pulse length on embryo developmental metrics.

Time of electroporation (hpi)	Pulse length (ms)	Replicates (r)	Zygotes (n)	Survived (n)	Cleaved (n)	Blastocysts	
						n	Mean ± SD %
10 h	1	4	226	202	159	81	35.84% ± 5.21%^a^
10 h	2	4	224	182	151	55	24.55% ± 3.98%^b^
10 h	3	4	226	177	138	48	21.24% ± 3.58%^b^
	Total	12	676	561	448	184	27.22% ± 3.78%^A^
18 h	1	4	266	245	214	103	38.72% ± 5.94%^a^
18 h	2	4	277	254	211	85	30.69% ± 5.27%^ab^
18 h	3	4	329	276	227	85	25.84% ± 4.66%^b^
	Total	12	872	775	652	273	31.31% ± 4.88%^A^

Zygotes were electroporated after 10 or 18 h using 5 pulses of 1, 2 or 3 ms at 25 V. Lowercase letters denote significant differences between pulse lengths (p < 0.05) within voltage groups. Uppercase letters denote significant differences within HPI groups.

*Hpi* hours post insemination.

^a,b,c^p < 0.05, ^A,B^p < 0.05.

### Edit event rates of developed blastocysts

**Figure 1:**
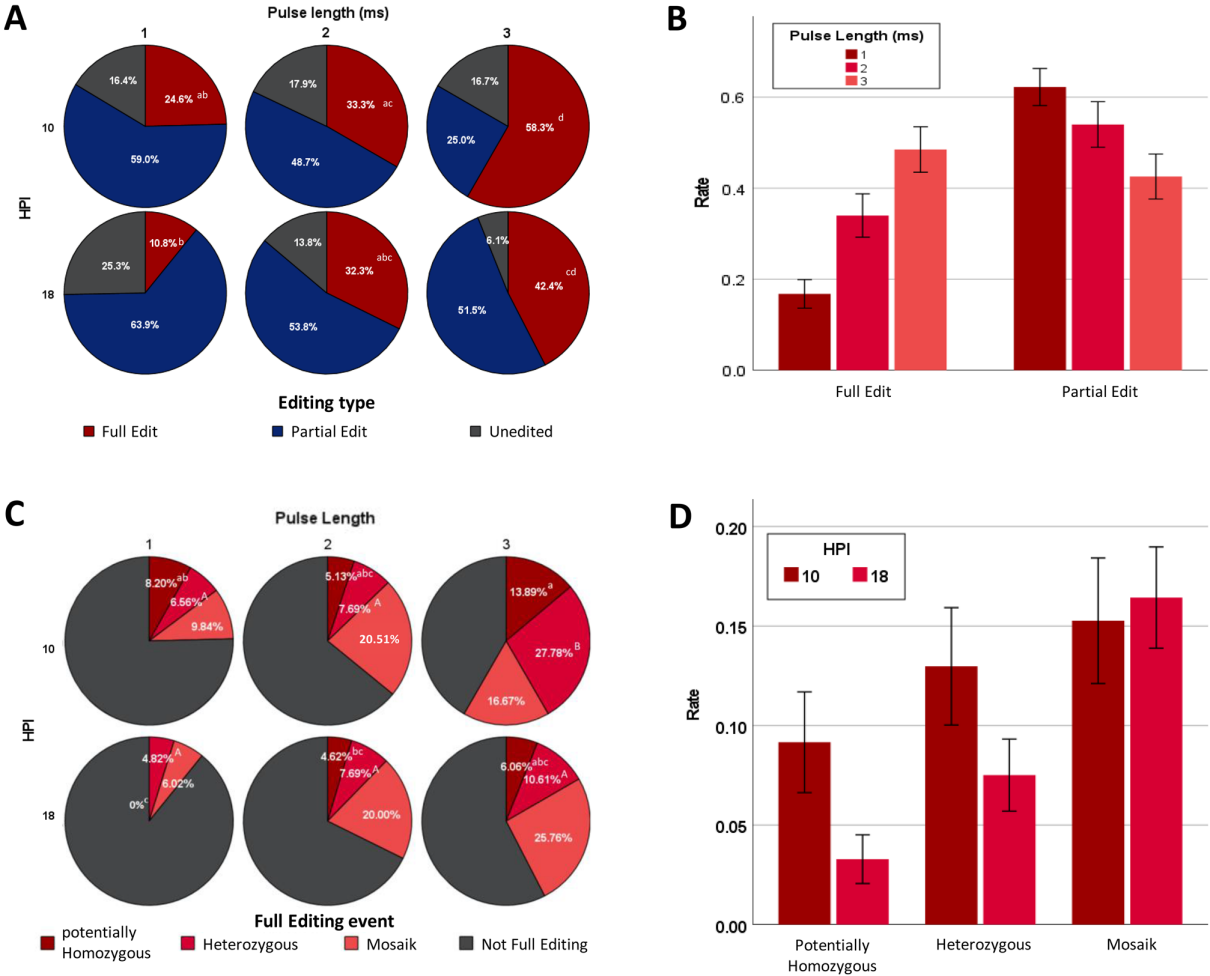
(**A**) Distribution of editing events under different pulse lengths and HPI. Pulse lengths of 3 ms (n = 36 blastocysts analyzed) resulted in significantly higher rates of Full edit (p < 0.05, lowercase letters) compared to 1 ms (n = 60 blastocysts analyzed) when electroporation takes place 10 HPI, respectively. (**B**) Solely effect of pulse length on rate of full and partial edits after combining groups electroporated at different times to one group. Rate of Full edits (encompassing heterozygous, potentially homozygous and mosaic edits) was significantly higher (p < 0.05, lowercase letters) for pulse lengths of 3 ms (n = 101 analyzed) compared to 2 ms (96 blastocysts analyzed) and 1 ms (n = 143 blastoysts analyzed) whereas rates of partial edits decreased with longer pulses vice versa. (**C**) Effect of Pulse length and Electroporation time (HPI) on distribution of edit events among Full edits of the same set of embryos. Pulse length of 3 ms (n = 36 blastocysts analyzed) resulted in a significantly higher rate of Heterozygous Full edits (p < 0.05, uppercase letters) compared to a Pulse length of 1 (n = 60 blastocysts analyzed) or 2 s (n = 35 blastocysts analyzed) when electroporation takes place 10 HPI, respectievely. Electroporation 18 HPI (n = 83 blastocysts analyzed) resulted in significantly lower rates of potentially Homozygous Full edits (p < 0.05, lowercase letters) compared to 10 HPI (n = 60 blastocysts analyzed) for Pulse durations of 1 ms. (**D**) Effects of early and late electroporation (10 vs. 18 HPI) on the rates of potentially homozygous, heterozygous and mosaic Full edits. Early electroporation (n = 131 blastocysts analyzed) resulted in significantly higher rates of potentially homozygous Full edits as well as heterozygous Full edits (p < 0.05,*) compared to late electroporation (n = 213 blastocysts analyzed) but had no effect on rate of mosaic Full edits.

A representative proportion of approximately 80% of all blastocysts derived from contrasting treatment groups were analysed for edit events (Fig. 1A). In total 131 blastocysts were Sanger sequenced representing those electroporated with pulse lengths of 1, 2 and 3 ms at 10 h hpi (n = 60, n = 35, n = 36, respectively). Likewise, a total of 214 blastocysts representing those electroporated (1 ms, 2 ms, 3 ms) at 18 hpi were analysed the same way (n = 83, n = 65, n = 66, respectively).

### Pulse length significantly affects edit event rate

In total, 335 blastocysts were analysed for edit events (Fig. 1A). Increasing pulse lengths (PL) significantly increased the Full Edit rate (p = 0.01) independently of time of electroporation, with the highest rates of Full Edits observed when zygotes had been electroporated 10 hpi and with 3 ms pulses (58.3 ± 15.7%) and the lowest when electroporated 18 hpi with 1 ms (10.8 ± 4.3%). Conversely, time of electroporation post insemination did not affect the proportion of Full Edits in general (Fig. 1A). Consequently, blastocysts electroporated at contrasting hpi were condensed into groups revealing again that higher pulse lengths (3 > 2 > 1) significantly increased the rate of full edits and decreased the proportion of partial edits between each PL (Fig. 1B).

### Time of electroporation post insemination affects full edit type

Mixed model analysis revealed that heterozygous edit rates were significantly affected by Pulse length and hpi (Table 2). The rate of heterozygous edits in zygotes electroporated with pulse lengths of 3 ms was significantly higher than those electroporated with 1 or 2 ms when electroporated 10 hpi (27.78, 7.69% and 6.6% respectively). Likewise, the rate was significantly higher when zygotes were electroporated with pulse lengths of 3 ms vs. 1 ms 18 h post insemination (10.6% and 6.0%, respectively) (Fig. 1C).

**Table 2:** Factor analysis of editing outcomes.

	Homozygous KO	Heterozygous KO	All non-mosaic KO	Mosaic KO	All KO	All mosaic
HPI	0.027*	0.046	0.002**	0.829	0.024*	0.152
PL	0.141	0.002**	< 0.001***	0.005**	< 0.001***	0.016*
HPIxPL	0.458	0.094	0.079	0.342	0.67	0.004**

Analysis of effect of experimental factors on knockout type: GLMM of HPI, pulse length and their interaction on rate of homozygous, heterozygous and mosaic knockouts. Values are p values of each factor for each variable.

*, ** and *** denote p values < 0.05, < 0.01 and < 0.001, respectively.

When comparing the effect of contrasting pulse lengths and hpi for their effect on type of full edits, mixed model analysis (Table 2) revealed that potentially homozygous edit rates were significantly affected by pulse length. Significantly higher rates of potentially homozygous edits were obtained for pulse lengths of 3 ms compared to 2 ms or 1 ms when both had been electroporated 10 hpi (13.9%, 5.13% and 8.2% respectively). Conversely, no effect of pulse length was observed when comparing the effect of pulse lengths on zygotes electroporated 18 h hpi (Fig. 1C).

Considering both pulse length as well as time of electroporation, the highest rates of both heterozygous and homozygous full knock outs were observed in the 10 HPI, 3 ms groups (13.9%) and the lowest in the 18 HPI 1 ms group (0%) as presented in Fig. 1C. When different PL groups were combined, a significant difference could be seen between 10 and 18 HPI in terms of both homozygous and heterozygous edit rates (Fig. 1D).

**Figure 2:**
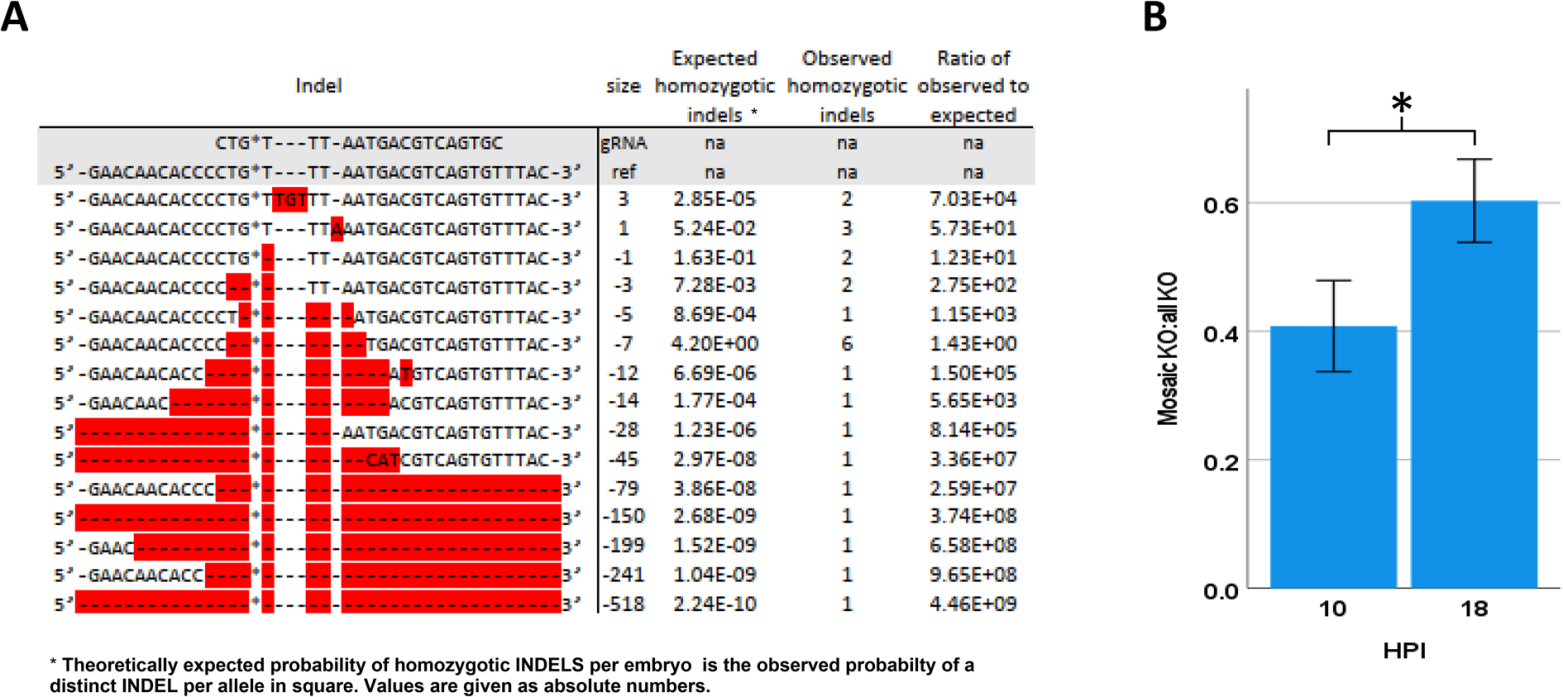
(**A**) Sequence details of all Homozygous Full edit editing events (n = 21) represented by a total of 15 INDEL editing variants. The probability of Loss-of-heterozygosity for of all these 15 INDEL variants involving both homologous chromosomes is far above expected plausibility based on likelihood of observed INDELS in single chromosomes suggesting a distinct mechanistic cause rather than random effects. (**B**) Effects of early (n = 131) and late (n = 213) electroporation (10 vs. 18 HPI) on mosaic edit to all edit ratio. Earlier electroporation resulted in a significantly lower mosaic edit to all edit ratio compared to later electroporation. *Denotes significant difference (p < 0.05) between groups.

Considering homozygous full Edits, a total of 15 INDEL variants could be detected (Fig. 2A). The probability of all these 15 INDEL variants involving both homologous chromosomes was far above expected plausibility based on likelihood of observed INDELS in single chromosomes. Moreover, these INDELS were randomly distributed. No difference was detected in overall Mosaic edits. However, the ratio of Mosaic edits to all full edits (Fig. 2B) was significantly lower in the 10 HPI groups.

### Time of electroporation post insemination affects mosaic edit per all edit ratio

The rate of mosaicism was significantly affected by PL (p = 0.016) as well as the interaction between PL and hpi (Table 2). While time of electroporation did not affect the proportion of Mosaic Full Edits per zygote (Table 2, Fig. 1C,D), increasing pulse lengths significantly increased proportions of Mosaic Full Edits both for zygotes electroporated 10 hpi (9.8%, 23.0% and 16.7%, respectively) as well as for zygotes electroporated 18 hpi (6.0% and 20.0%, 25.8%, respectively). In contrast, when calculated relative to the proportion of Full edit embryos, aggregated groups of zygotes electroporated at 10 hpi ended up with a significantly lower Mosaic edit to all edit ratio than those electroporated 18 hpi (Fig. 2B).

### Characteristics of loss-of-heterozygosity events on chromosomal level

**Figure 3:**
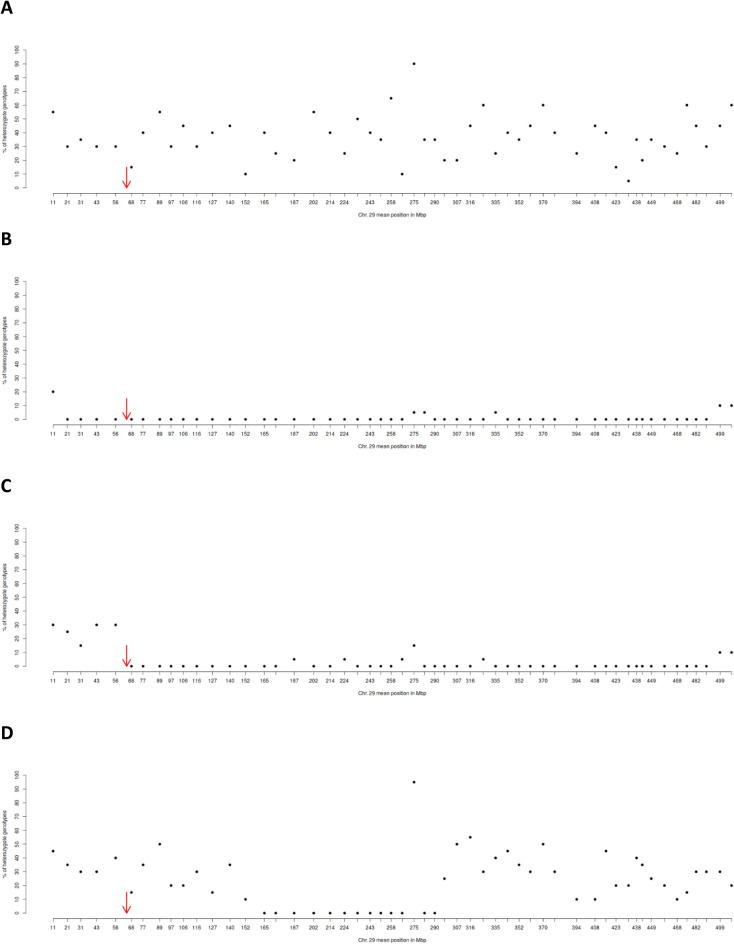
Representative graphical outline of embryos identified to represent homozygous full edits. Proportion of SNP-heterosygosity along chromosomes 29 is reported for DNA-windows with a red arrow indicating the Cas9 target site. Each spot represents state of heterosygosity of one window encompassing a total of 20 SNP-marker. All in all 11 out of 23 embryos showed no Loss-of-heterozygosity in chromosome 29, containing the target side, and were therefore classified as non-disrupted as demonstrated exemplarily in (**A**). Conversely, 1 embryo showed Loss-of-heterozygosity along the entire target-side arm of the chromosome and were therefore classified as Loss-of-chromosome (**B**) whereas 3 embryos displaying losses of heterozygosity distal the target site were classified as Loss-of-chromosomal arms (**C**). A total of 6 Embryos displaying Loss-of-heterozygosity along large segments within the chromosome were classified as Loss-of-chromosomal section (**D**).

A total of 23 embryos identified to represent homozygous full edits were genotyped using SNP analysis and analysed for heterozygosity along the entire genome to further analyse the cause for their Loss-of-heterozygosity (Table 3). Of the 23, 2 showed complete Loss-of-heterozygosity in every chromosome as shown in Supplemental Fig. 2. These were classified as being Loss-of-diploidy. Clearly, embryos showed less mean heterozygosity with regard to chromosome 29. Of the remaining 21 embryos, 11 showed no Loss-of-heterozygosity in chromosome 29, containing the target side, and were therefore classified as non-disrupted as demonstrated exemplarily in Fig. 3A. Conversely 1 embryo showed Loss-of-heterozygosity along the entire chromosome 29 and was therefore classified as Loss-of-chromosome (Fig. 3B) whereas 3 embryos displaying losses of heterozygosity distal the target site were classified as Loss-of-chromosomal arms (Fig. 3C). Finally 6 Embryos displaying Loss-of-heterozygosity along large segments within the chromosome were classified as Loss-of-chromosomal section (Fig. 3D). Thus, a proportion of 47.8% of all embryos found to display Loss-of-heterozygosity at the target site showed no indication of Loss-of-chromosomal sections whereas a proportion of 52.2% were identified to suffer from entire or sectional Loss-of-chromosomal regions.

**Table 3:** Rationale of Loss-of-heterozygosity of embryos showing homozygous biallelic editing.

Embryo no.	Loss-of-heterozygosity	Loss-of-diploidy	Loss-of-chromosome	Loss-of-chromosomal arm	Loss-of-chromosomal section
1	+	−	−	−	+
2	+	−	+	−	−
3	+	−	−	−	−
4	+	−	−	−	−
5	+	−	−	−	+
6	+	−	−	−	−
7	+	−	−	−	−
8	+	−	−	−	−
9	+	−	−	−	−
10	+	−	−	+	−
11	+	−	−	−	−
12	+	−	−	−	−
13	+	+	−	−	−
14	+	−	−	+	−
15	+	−	−	−	+
16	+	−	−	−	−
17	+	−	−	−	+
18	+	−	−	−	−
19	+	+	−	−	−
20	+	−	−	−	−
21	+	−	−	+	−
22	+	−	−	−	+
23	+	−	−	−	+
Total		2	1	3	6

Classification of embryos (n = 23) identified to represent homozygous full edits based on Loss-of-heterozygosity pattern as determined by proportion of SNP-heterozygosity considering subsequent DNA windows. Of these embryos, 2 showed complete Loss-of-heterozygosity in every chromosome and were classified as being Loss-of-diploidy. Of the remaining ones a total of 11 embryos were classified as non-disrupted. Conversely 1 embryo was classified as Loss-of-chromosome, 3 embryos were classified as Loss-of-chromosomal arms and 6 Embryos were classified as Loss-of-chromosomal section. Details of all embryos can be checked in Suppl. Fig. 3.

## Discussion

In this study, we analysed the biological parameter of cell zygote cycle stage in combination with variations of the technical setting pulse duration in the context of Cas9 RNP-electroporation. This was done to enhance our basic understanding about relevant factors affecting characteristics of genome editing outcome and to deepen our knowledge about the mechanism of distinct editing events in bovine embryos. In accordance with previous findings conducted in species like mice, rats and pigs, this study shows that Cas9 RPN electroporation is a highly efficient method for editing of embryos^22,23^ while keeping high developmental rates. The overall editing efficiency was determined based on the number of edited blastocysts. Noteworthy, base editing efficiency of blastocysts was not significantly affected by any of the factors under study, showing rates around 80% or more, and comparable with efficiencies reported by previous studies in human^24^, mouse^15^, and other animal models^12^.

Here, our results indicate that 25 V is efficient for editing of bovine zygotes. Indeed, another study involved in electroporation of bovine zygotes used lower voltages also admitted in the murine model^17^. In that study, however, editing efficiencies were not analysed for bovine zygotes electroporated with RNPs nor was the effect of contrasting pulse durations or timepoints. So far only one study has investigated the effect of time of injection of mRNA encoding the Cas9 protein as well as the required gRNA into bovine zygotes. In agreement to the present study, the authors of that study speculated that earlier injections might increase overall editing efficiencies, although direct comparability of these two studies might be limited^19^. Distinct consequences for editing characteristics because of contrasting timepoints however, have not been analysed nor reported.

Of high interest, the results of the present study uncovered that increasing pulse lengths significantly increases the Full Edit rate and concurrently reduced the proportion of Partial Edits when embryos were pulsed with 25 V independently of time of electroporation. This interdependence between pulse duration and Edit event rate has been suggested to some extent in earlier studies while not yet being investigated for bovine zygotes^13,15,23^. Presumably, longer pulses allow more RNPs to bypass a zygote’s cell membrane, increasing the amount of Cas9 activity, and thereby increasing the chances of DNA cleavage. Although longer pulses did lead to lower development rates, the higher rate of full edits resulted in a larger quantity of blastocysts with these desired edits.

In depth investigation of Full Edit types, discriminating between homozygous, heterozygous and Mosaic Full Edits, showed a significant relationship between these and contrasting pulse lengths and times of electroporation. The proportion of heterozygous full Edit types was significantly affected by pulse length and time of electroporation. Highest rates for heterozygous Edits, accounting for nearly 30%, were obtained when zygotes were treated 10 hpi with 3 ms pulse length with much lower values for 2 ms and 1 ms. In agreement, the present study revealed that the proportion of homozygous Full Edits was significantly affected by pulse length when electroporated 10 hpi, with longer durations being more advantageous than shorter ones. Conversely, no effect of pulse length was obtained for homozygous Full Edit type when zygotes were electroporated 18 h hpi. This different effect of contrasting times for electroporation became even more obvious when combining different pulse lengths into one group. Taken together, representing one of the main outcomes of the present study, time of electroporation significantly affected the proportion of both homozygous and heterozygous full Edits, with electroporation at 10 hpi clearly promoting the creation of homozygous full Edits, accounting for nearly 15%. This has been shown in other animal models as well, some using inter cytoplasmic sperm injection to introduce Cas9 RNPs at the moment of fertilization^24^, but has not been shown in the bovine system, yet.

Keeping in mind that earlier studies have proved that in vitro produced bovine zygotes will start chromosomal replication, resembling onset of S-Phase, at the earliest at 10 hpi with nearly all of them having finished chromosomal replication 18 h after onset of IVF^21^, the present study demonstrates that timing of electroporation relative to a zygote’s cell cycle stage clearly affected homozygous biallelic editing of bovine zygotes. In other words, Cas9 RNP-electroporation prior to onset of S-Phase resulted in much higher rates of homozygous biallelic editing events compared to electroporation after S-Phase. Given that the observed frequency of identical editing involving both homologous chromosomes is far above the expected frequency based on the likelihood of observed INDELS, it becomes clear that a distinct mechanism is a requisite for such an outcome.

In Drosophila, CRISPR-induced double-strand breaks triggering recombination between homologous chromosome arms have been reported^25^. Keeping in mind that CRISPR induced DNA double-strand breaks are repaired by either error-prone non-homologous end joining (NHEJ) or by homology direct repair (HDR), homozygous biallelic editing would require one edited homologous chromosome, having previously undergone NHEJ, serving as a template for HDR of its sister chromosome.

Even earlier, Sadhu and colleagues induced a single double strand break in one chromosome of a diploid yeast strain and achieved homologous recombination-based “Loss-of-heterozygosity” events at the target site^26^. Other reports suggested that homologous recombination-based “Loss-of-heterozygosity” is not restricted to animals but is also observed in tomatoes^27^. With respect to future breeding programs in bovine, CRISPR based Loss-of-heterozygosity of desired genes of interest might indeed be an attractive option enabling the generation of offspring homozygous for a given allele of interest, while only inheriting that allele uniparentally. This explanation is in accordance with a recent study reporting that donor templates for HDR might be located elsewhere on the chromosome, intrachromosomal on the sister chromatid (intrachromosomal HDR) or on the homologous chromosome (interchromosomal HDR)^28,29^. Although the homologous repair machinery exhibits a strong preference for intrachromosomal donor templates given a choice between two available donor templates, interchomosomal homologous recombination is possible and occurs as frequently as intrachromosomal HDR^29^. Some years ago, it was reported that controlled timing of CRISPR/Cas9 delivery increased homology-directed repair in human cultured cells, showing the highest efficiencies when delivered in M phase^30^. While that cell-cycle stage closely resembles our groups of zygotes electroporated after S Phase (18 hpi), HDR has been reported to already occur during S/G2 Phase^28^. That could explain why Cas9 RNP-electroporation of zygotes 10 hpi, therefore before onset of S-phase with resulting absence of the sister chromatid, induces INDELS which can only be repaired by the homologous repair machinery via the homologous interchromosomal template. In accordance, donor-free gene correction by targeted interhomolog recombination has been reported a frequent event after double strand breaks targeted by CRISPR/Cas9 in cultured human cells during G2 phase but not G1 phase^31^. The mechanism by which this homology search takes place, however, is largely unknown.

In the bovine, Loss-of-heterozygosity because of homozygous biallelic edits showing identical INDELS on both homologous chromosomes, have not been reported via CRISPR-Cas9 induced genome editing in bovine. Strikingly, the possibility of correction of pathogenic mutations through interhomolog recombination with a lack of mosaicism, would have major advantages over other approaches, given that it does not require introduction of exogenous nucleic acids, limiting it to alleles already in the cattle population and would therefore be highly attractive for breeding schedules. Although very recent results in drosophila have shown that a substantial amount of CRISPR-Cas9-induced double strand breaks result in exchange between homologous chromosome arms^25^, and even more that these occur in multiple experimental settings and not only under specific conditions, one should not neglect, however, the option that seemingly homozygous edits, appearing as only one INDEL within the target region without any detectable wildtype copy after sequence analysis, could also be a consequence of Loss-of-chromosomal material or even entire chromosomal arms proximal or distal to the targeted locus^32,33^.

Indeed, Zuccaro and colleagues reported that a considerable proportions of double strand breaks induced by CRISPR Cas9 in human oocytes remained unrepaired, resulting in undetectable alleles^32^. Similarly, upon reanalysis of embryos from previous work, Alanis-Lobato and colleagues found that 16% of embryos had unintended editing events around the target site including segmental loss and gain of the chromosome^33^.

Therefore, the present study aimed to discriminate Loss-of-heterozygosity events caused by loss of entire chromosomes, Loss-of-chromosomal arms, or large chromosomal intersections.

Therefore DNA of 23 embryos confirmed to represent potentially homozygous editing were further analysed by SNP typing to compare section specific heterozygosity for chromosome 29 harbouring the target site. Interestingly, 2 out of 23 embryos showing Loss-of-heterozygosity on chromosome 29 were found to be entirely haploid. Development of bovine oocytes to blastocysts due to parthenogenetic activation and thus without complementation of the diploid genome has been reported to occur after electrical stimulation^34^. Nonetheless we were surprized that RNP-electroporation activated unfertilized oocytes, which underwent cellular DNA repair and INDEL formation, and developed into morphologically normal blastocysts.

Furthermore, chromosomal SNP analysis and subsequent in-depth investigation of chromosomal SNP heterozygosis in the present study shows that roughly 1 out of 2 embryos showing potentially homozygous editing displayed Loss-of-heterozygosity that suggests large deletions or losses of whole chromosome arms or sections. Among these, 1 out of 21 embryos displayed complete Loss-of-heterozygosity with respect to Chromosome 29. Thus, it is very likely that one entire Chromosome 29 got lost in these embryos. This is in agreement to a recent study reporting whole chromosome loss in mouse embryos^35^ as well as in human embryos^33^ after genome editing. Likewise, 3 out of 21 embryos displayed Loss-of-chromosomal arms. This has been reported very recently in mouse embryos after CRISPR Cas9 editing^35^ and earlier using cell lines^36^. It has been speculated that once a Cas9-mediated double strand break takes place it results in two fragments, one of which contains a centromere (centric fragment) and another one does not (acentric fragment). If unrepaired by the time of mitotic entry, the acentric fragment is prone to chromosome missegregation resulting in segmental chromosomal loss^35,37^. Finally, 6 out of 21 embryos displays loss of chromosomal intersections with some of them without a direct relationship to the target site. This is of great concern since edited embryos may carry the homozygous INDEL of interest while large scale loss of heterozygosity at distance from the target site may remain undetected and impact viability and/or phenotype of independently of the locus of interest. In that context, our strategy to check for genome wide sectional loss of heterozygosity might be a useful strategy to identify loss of chromosomal intersections without direct relationship to the target site.

The cause for these unrelated intersectional losses, however, is unclear. While we were able to exclude the presence of a potential additional off-target site of our guide RNA on chromosome 29, it cannot be fully excluded that this might represent a technical artefact caused by “allele drop-out” taken place during foregone whole genome amplification. Also these intersectional losses might have been initially very well related to a large deletion at the target site. While regions in close proximity to the target site might have been repaired by HDR whereas loss of chromosomal segments at distance from the target site persisted. Thus even it seems that chromosomal deletions appear to be not related to the target site it cannot be excluded at all that they have hat a relation initially. In great accordance, breakpoints of chromosomal arms on a given chromosome have been reported to be independent in some cases from the target site on that chromosome even earlier in the mice^35^ while this was not reported for bovine embryos so far.

In the present study nonetheless, 1 out of 2 embryos demonstrating Loss-of-heterozygosity at the target site showed a non-disrupted chromosome 29 with normal SNP-heterozygosity around the target site. These results suggest that some of the Loss-of-heterozygosity events are indeed caused potentially by homozygous editing events. That possibility has been proposed before^24^. The authors proposed that in edited embryos one allele served as a template for the homology directed repair pathway to repair the double strand break^24^. The results of our analysis based on heterozygosity within chromosomal sections are also in agreement with a recent study aiming to answer the same question by comparing the gene expression profile of genes in chromosomal proximity to the target site of human embryos showing loss-of-heterozygosity, reporting that 68% of CRISPR-Cas9 targeted cells did not exhibit any obvious segmental or whole chromosomal abnormality^33^. In that light, the present study would be the first demonstration of homozygous editing in bovine because of interchromosomal recombination. Apart from that interpretation, however, cannot neglect the theoretical possibility that some of these embryos demonstrating Loss-of-heterozygosity, although bearing non-disrupted chromosomes, might contain large sequence deletions overriding PCR primers and therefore resulting in escape of PCR amplification^3,37–40^. Keeping in mind the enormous potential of homozygous editing due to interchromosomal HDR for breeding purpose in the bovine, confirmation of its rate of occurrence and further optimization of the rate of interchromosomal repair and concurrent reduction of the occurrence of chromosomal damage should be a major focus of research in order to enhance the applicability of genome editing technology. Consequently, further studies are necessary to determine how broken chromosomes are repaired to exclude Loss-of-chromosomal arms or even entire chromosomes prior to use in reproduction.

Supplementarily, our findings show that embryos derived from zygotes either electroporated before S-phase (10 hpi) or after S-phase (18 hpi) showed highly comparable relative distributions of editing events (Suppl. Fig. 1). Noteworthy, identical deletions of 7 bp flanked by homologous TG sequences were highly overrepresented. This is in agreement with similar natural mutations observed in vivo^41,42^ and induced in gene editing experiments^32^. This reinforces the observation that INDEL profiles can be non-random depending on target^43^. Moreover, the results of the present study are in accordance with a recent study which showed target-specific prediction of INDELS using CRISPR-mediated genome editing^44^. Indeed, precision of DNA editing was reported to vary considerably among targets, but some targets showed one highly preferred INDEL depending mainly on the fourth nucleotide upstream of the protospacer^44^. This supports our suggestion that distinct editing events are a consequence of target specific factors like nucleotide sequence and chromatin status but not a result of timing of RNP-electroporation to a zygote’s cell cycle stage.

Finally, the present work highlights the relevance of the time of electroporation for the occurrence of mosaic-edits resulting in much higher rates of mosaicism among full knock out embryos when RNP-electroporation took place after S-phase. Generally, mosaicism occurs when editing happens after the first DNA replication of the zygote, meaning that: DNA repair mechanisms failed to induce an edit before S-phase, those edits were not different enough from the wildtype to prevent further Cas9 activity after replication, or introduction of the Cas9 RNPs after S-phase. The first two sources of failure can likely be solved by increasing Cas9 nuclease activity, through increasing cytoplasmic RNP concentration via increased voltage or pulse length as underlined by the results of the present study. The third can only be addressed by earlier introduction of Cas9 into the zygote. Comizzoli and colleagues have shown that most in vitro produced bovine zygotes will have undergone their first replication before 18 h after IVF, meaning that earlier electroporation is likely necessary to reduce mosaicism and induce biallelic editing^21^. Indeed, while editing rates in general were not significantly different between electroporation times, in terms of the type of editing induced, early electroporation reduced the rate of mosaicism. At this point however one has also keep in mind that sequence trace decomposition after Sanger sequencing (TIDE) remains a low percentage of mosaicism undetectable^45^. Consequently, the real rate of embryos fraught with mosaicism might be even slightly higher whereas the general outlines of our study should not be affected by that technical limitation. When factoring in mosaic embryos that were nonetheless full edits, electroporating after 10 h was even more efficient at inducing desired edits. This is also most likely due to the lower quantity of gene copies when electroporating before S-phase, as the likelihood of knocking out all wild-type copies decreases as the copy number increases and the nuclease is diluted among daughter cells. These effects compounded with increased pulse length. While editing rates remained relatively stable, the ratio of biallelic to mosaic edits steadily increased with pulse length. Presumably, longer pulses allow more RNPs to bypass the cell membrane, increasing the amount of Cas9 activity, and thereby increasing the chances of earlier DNA cleavage. In terms of the precise repair of deleterious variants or introduction of desired traits, biallelic edits would be candidates for precise gene editing using donor templates, as mosaic animals are unlikely to find acceptance in cattle breeding, where breeding out a homozygote would take years.

Altogether, the present study confirms that Cas9 RNP-electroporation is an attractive method to edit bovine zygotes. Of high impact, the present study shows that increasing pulse lengths generate higher full edit rates as well as higher proportions of full edit embryos showing loss-of-heterozygosity when electroporated prior to S-phase. Likewise, the present study shows that mosaicism as well as loss-of-heterozygosity in full-edit embryos is clearly related to timing of electroporation relative to a zygote’s cell cycle stage, with electroporation prior to onset of S-phase being more beneficial. Loss-of-heterozygosity is reported for the first time after genome editing in bovine embryos. Probability of Loss-of-heterozygosity events in turn was related to timing of electroporation relative to a zygote’s cell cycle stage. The present study draws attention on the frequent generation of chromosomal alterations accounting for approximately 50% of all Loss-of-heterozygosity events. Occurrence of large scale losses even at huge distance from the target site, as observed in this study, is of great concern and should be further considered in future studies before this technique can be used in bovine reproduction.

A high proportion of these loss-of-heterozygosity events, however, was shown not to be a consequence of chromosomal breaks and therefore potentially a result of interhomolog recombination, a mechanism which offers a very attractive option for future breeding schedules and correcting pathogenic mutations without requiring the introduction of exogenous nucleic acids. While nearly 50% of all loss-of-heterozygosity events are not a consequence of loss of larger deletions surrounding the target site, it also cannot be fully excluded that some are identified wrongly due to loss of PCR primer sequences after INDEL formation. Therefore, further studies should be conducted to determine the relative occurrence of interhomolog recombination before this technique can be used in bovine reproduction.

## Materials and methods

### Experimental design

This study was designed as a 2 × 3 factorial model. Specifically, the zygotes were electroporated after 10 or 18 h of fertilization, at 25 V, using 1, 2 or 3 ms pulses. All other factors were kept constant. Each condition was tested in 4 replicates.

### gRNA and primer design

**Figure 4:**
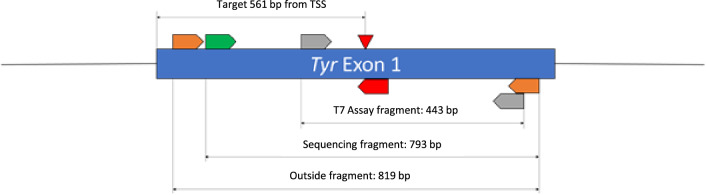
Target and PCR primer schematic. Red elements show gRNA and cut site position. Orange, grey and green elements show nested PCR primer positions.

The Benchling online software tool (Benchling) was used to design both the gRNAs and PCR primers. PCR Primers were then verified using NCBI’s Primer-Blast Tool and the PCR Primer Stats Tool. Exon 1 of *Tyr* was targeted for editing (Exon 1, 561 (−), GCACTGACGTCATTAAACAG, PAM ggg). Targets were selected based on on-target efficiency and off-target scores. In the end one target, with a cleavage site at bp 561 from the initial ATG was used for the experiment (Fig. 4). PCR primers were designed for nested PCR, amplifying an initial outer fragment. Primer sequence and position can be found in Supplementary Table 1.

### In vitro production of bovine Zygotes

Recipes for culture media can be found in supplementary materials 1. Cumulus-oocyte-complexes (COC) were harvested from ovaries collected from a partner slaughterhouse (Bernhard Frenken GmbH, Düren, Germany). Non-dominant follicles were aspirated with 18 gauge needles and syringes. Morphologically normal, immature COCs with tightly bound cumulus cells at least 4 layers deep were collected and transferred to TCM air. They were then washed three times in maturation medium before being transferred to 4 well plates (NUNC) in groups of c. 50 COCs/well, with 400 μl of maturation medium per well, each topped with 400 μl of mineral oil. They were then incubated for 22 h at 39 °C with 5% CO_2_ and backfilled with air. The matured COCs were then washed in fertilization medium and coincubated with capacitated, swim-up derived sperm in 400 μl of fertilization medium with 2 mio. sperm/ml. Coincubation (39 °C, 5% CO_2_, backfilled with air) lasted either 10 or 18 h.

### Cas9 RNP-electroporation of zygotes

To prepare the crRNA/tracrRNA duplex, crRNA and tracrRNA (IDT) were diluted to a concentration of 100 μM in T_10_:E_1_ buffer (10 μM TRIS–HCL, pH 7.5, 1 μM EDTA). 3.75 μl of each was then combined with 2.5 μl T_10_:E_1_ buffer in a PCR tube and heated to 95 °C for 5 min and then cooled at a rate of 6 °C/min to 4 °C. The duplex solution was held at 4 °C until needed.

After either 10 or 18 hpi, presumptive zygotes were washed in TCM air, transferred to 1 ml TCM air in a 15 ml tube, and vigorously vortexed with a counterweight to remove the cumulus cells. Cells were then sorted into groups and transferred into wells with 400 ml of SOF media overlaid with oil, and incubated (39 °C, 5% CO_2_, backfilled with air) until ready for electroporation. The electroporation media was prepared concurrently. 1.6 μl crRNA/tracrRNA duplex was combined with 2 μl TCEP buffer, 0.975 μl of 10 μg/μl Cas9 protein (IDT), 5.425 μl T_10_:E_1_ buffer and 10 μl OptiMEM. The final concentration of RNP was 3 μM. The electroporation media was then held warm for at least 10 min on a heated laboratory bench for embryo culture, never exceeding 37 °C. Zygotes were then washed in 2 consecutive 200 μl drops of OptiMEM media and then transferred to 20 μl of electroporation media. Zygotes and electroporation media were then transferred to 1 mm gap cuvettes and electroporated at according to the separate experimental conditions 25 or 30 V for 5 pulses of either 1, 2 or 3 ms. Zygotes were then removed from the cuvettes and collected in 400 ul of OptiMEM and then washed in 3 consecutive 200 μl drops of SOF media before being returned to culture plates. The zygotes were then cultured (39 °C, 5% CO_2_, 5% O_2_, backfilled with N_2_). Survival and cleavage rates were determined on day 3 after electroporation and blastocyst rates were determined on day 8.

### Analysis of day-7 embryos

Embryo lysis and analysis was adapted from an earlier study^46^. Briefly, the blastocysts were removed from their culture medium and washed in PBS, transferred to PCR tubes with 5 μl millipore H_2_O and then stored at -20 °C. For lysis, 5 μl of 2 × lysis buffer (200 M Tris HCl [pH 8.3], 200 mM KCl, 0.008% Gelatin, 0.9% Tween, 100 μg/ml glycogen, 125 μg/ml Proteinase K) was added to each tube and then lysed overnight in a thermocycler (50 °C for 8 h, followed by 95 °C for 10 min and then held at 4 °C). Samples were the stored at -20 °C until further use.

Sample DNA was then amplified via nested PCR. PCR primers were ordered from Eurofins Genomics, while 10 × buffer and taq-polymerase was ordered form Genaxxon Bioscience. The initial PCR used 2 μl of sample material, with 0.5 μl of forward and reverse outside primers, 2 μl of 10 × Buffer, 0.5 μl DNTPs and 0.4 μl of taq-polymerase, then filled to 20 μl with bidest H_2_O. Cycler condition were: 95 °C for 3 min, then 35 cycles of 95 °C for 30 s, the primer temperature for 30 s, and 72 °C for 60 s, followed by a final elongation at 72 °C for 10 min. Round 2 of the nested PCR used the same conditions except that the sample volume was 1 μl of the round 1 product, and the inside primers and corresponding annealing temperature were used. Amplification was confirmed via gel electrophoresis, whereafter the samples were stored at -20 °C.

A subset of Blastocysts (~ 80% of each group) were then sequenced using Eurofins Genomics PlateSeq service for PCR products. 7.5 μl of sample was combined with 7.5 μl of bidest and delivered overnight. Sequencing results were then analysed using the TIDE software tool for analysis of sequence decomposition to determine INDEL sizes and quantity as reported elsewhere^45^. Sequences were cross-checked by visual comparison of sequence alignments created by the Benchling software alignment tool.

### Editing event classification

A representative subset of blastocysts (~ 80% of each treatment) was Sanger sequenced and analysed by sequence alignment and decomposition analysis to determine the distribution of editing events. Editing was classified as Full Edit in case of no presence of the wildtype allele, Partial Edit represented by presence of both at least one edit and one wildtype allele and Unedited (only wildtype alleles). More detailed, Full Edits were further classified as potentially Homozygous edit (a single detected edited allele), Heterozygous edit (unique edits in two detected alleles), Mosaic edit (greater than two unique alleles present without wildtype detected).

Conversely, embryos were also classified as non-mosaic edit (homozygous or heterozygous Full edit), Mosaic edit (greater than two unique alleles present without wildtype detected) and Incomplete (one edited allele and one wildtype allele).

### Analysis of loss-of-heterozygosity rationale

Extracted DNA of Embryos determined to potentially have homozygous editing according to Sanger sequencing were whole genome amplified using the REPLI-g Single Cell Kit from Qiagen following the manufacturer’s instructions and then genotyped using the EuroG_MDv2_HTS_DEU BeadChip on an Illumina iScan. SNPs with a call rate below 95% were removed from further analysis and remaining SNP data were then analysed for heterozygosity using the SNP analysis tool PLINK in R. All in all, 23′736 SNP markers were available for further analysis. In a first step the percentage of heterozygote genotypes indicating presence of two chromosomal segments with regard to that DNA region per embryo and chromosome was investigated. In a second step BTA 29 was investigated further. The proportion of heterozygote SNPs was calculated for a total of 53 bins each comprising 20 SNP-markers.

A scatter plot graph displaying the mean Mbp-positon of each of those subsequent windows on the x-axis and the corresponding proportions of heterozygosity of that window on the y-axis was plotted for each embryo based on these data. Embryos showing Loss-of-heterozygosity for all chromosomes were classified as being “Loss-of-diploidy” resembling a haploid genome. Embryos displaying no Loss-of-heterozygosity were classified as being “Non-disrupted”. Embryos showing Loss-of-heterozygosity for all windows of chromosome 29 were classified as “Loss-of-chromosome”. Embryos showing Loss-of-heterozygosity for all windows distal the target site on chromosome 29 were classified as “Loss-of-chromosomal arm”. Embryos showing Loss-of-heterozygosity for a segment encompassing a minimum of 3 subsequent bins inside chromosome 29 were classified as “Loss-of-chromosomal section”.

### Statistical analysis

Statistical analysis was performed using the SPSS 26 Software package. Statistical differences between experimental factors in Embryo development metrics were determined using a generalized linear mixed model (GLMM) where each electroporation group was the experimental unit, the factors HPI and pulse length were experimental factors, and IVF replicates were random effects. Editing event comparison was performed using a GLMM where each embryo was the experimental unit, HPI and pulse length were experimental factors and electroporation groups nested within IVF replicates were random effects. Pairwise comparisons between groups used Sidak correction for multiple comparisons. All graphics were created using SPSS, Excel and R.

### Ethics declarations

All methods were performed in accordance with the relevant guidelines and regulations. No experiments including live animals have been conducted.

## Supplementary Information

Supplementary Figure 1.

Supplementary Figure 2.

Supplementary Figure 3.

Supplementary Table 1.

## Data Availability

The datasets generated during the current study are available in the OpenScienceFramework (OSF) repository [https://osf.io/qad5j/?view_only=58a4800d0560457c8d9c36dc0490ebe2]. Analyzed data are included in this published article (and its Supplementary Information files).
