# Author Correction: The cell cycle stage of bovine zygotes electroporated with CRISPR/Cas9-RNP affects frequency of Loss-of-heterozygosity editing events

**DOI:** 10.1038/s41598-022-25117-1

**Published:** 2022-12-09

**Authors:** Dennis Miskel, Mikhael Poirier, Luisa Beunink, Franca Rings, Eva Held, Ernst Tholen, Dawit Tesfaye, Karl Schellander, Dessie Salilew-Wondim, Carina Blaschka, Christine Große-Brinkhaus, Bertram Brenig, Michael Hoelker

**Affiliations:** 1 Institute of Animal Sciences, Animal Breeding, University of Bonn, Endenicher Allee 15, 53115 Bonn, Germany; 2 Department of Animal Science, Biotechnology and Reproduction of Farm Animals, Georg August University Goettingen, Burckhardtweg 2, 37077 Goettingen, Germany; 3 Department of Molecular Biology of Livestock, Institute of Veterinary Medicine, Georg August University Goettingen, Burckhardtweg 2, 37077 Goettingen, Germany; 4 Department of Biomedical Sciences, Animal Reproduction and Biotechnology Laboratory, Colorado State University, 3105 Rampart Rd, Fort Collins, CO 80521 USA

Correction to: *Scientific Reports* 10.1038/s41598-022-14699-5, published online 24 June 2022

The original version of this Article contained an error in the spelling of the author Bertram Brenig which was incorrectly given as Brenig Bertram.

The original Article has been corrected.

