# Supplementary figures and images for: The cell cycle stage of bovine zygotes electroporated with CRISPR/Cas9-RNP affects frequency of Loss-of-heterozygosity editing events
Source: Sci Rep. 2022 Jun 24;12:10793. doi: 10.1038/s41598-022-14699-5 (PMC9232522; doi:10.1038/s41598-022-14699-5)

Miskel et al., Suppl. Figure 1

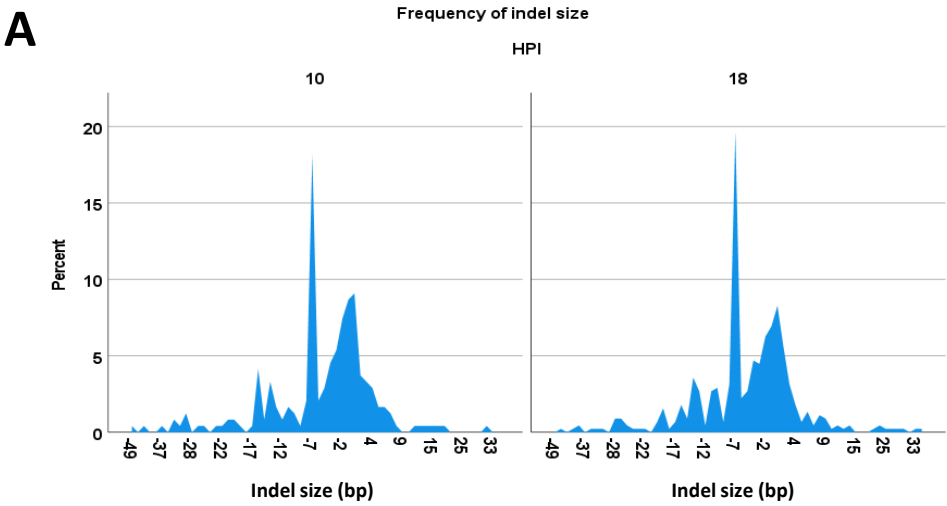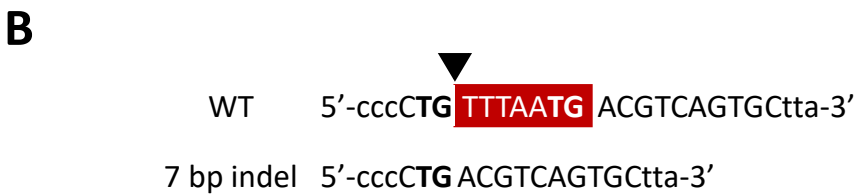

Supplement: Supplementary file 1 — Supplementary Figure 1. [file 41598_2022_14699_MOESM1_ESM.pdf]

Miskel et al. Suppl. Figure 2

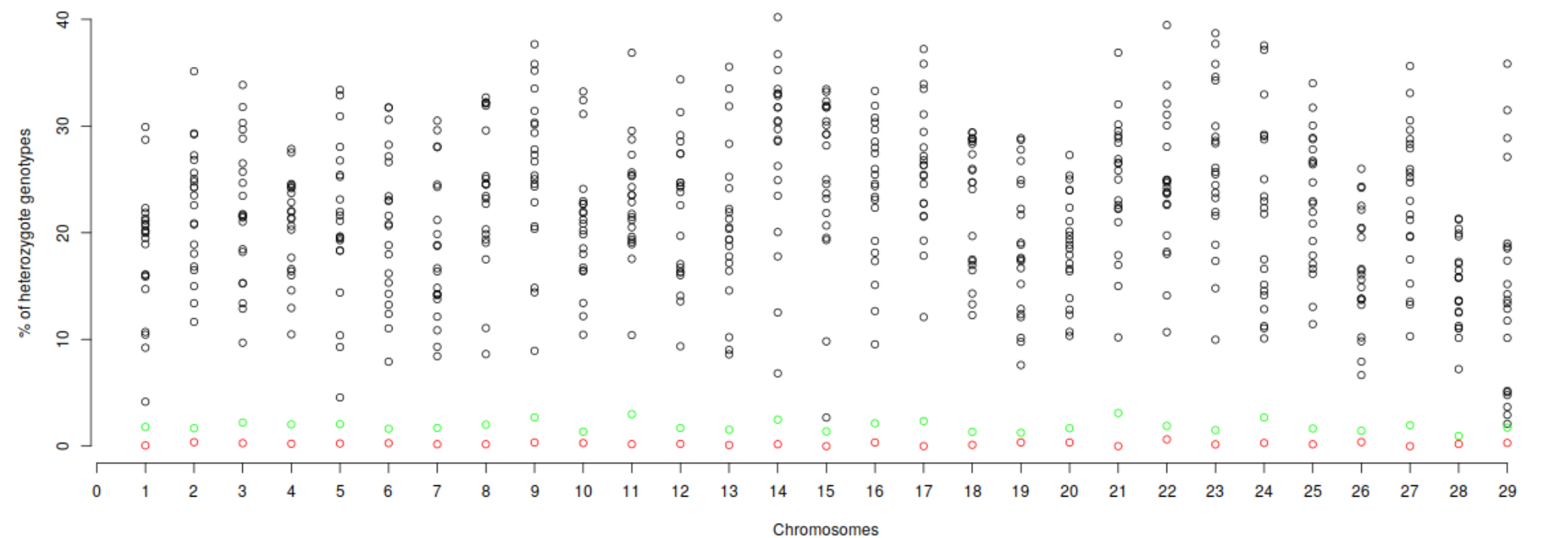

Supplement: Supplementary file 2 — Supplementary Figure 2. [file 41598_2022_14699_MOESM2_ESM.pdf]
