# Supplementary material for: The cell cycle stage of bovine zygotes electroporated with CRISPR/Cas9-RNP affects frequency of Loss-of-heterozygosity editing events
Source: Sci Rep. 2022 Jun 24;12:10793. doi: 10.1038/s41598-022-14699-5 (PMC9232522; doi:10.1038/s41598-022-14699-5)

Miskel et al. Suppl Figure 3

Embryo 1

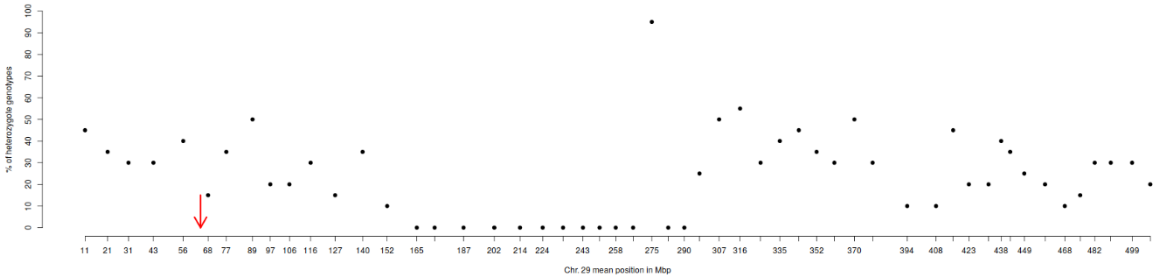

Embryo 2

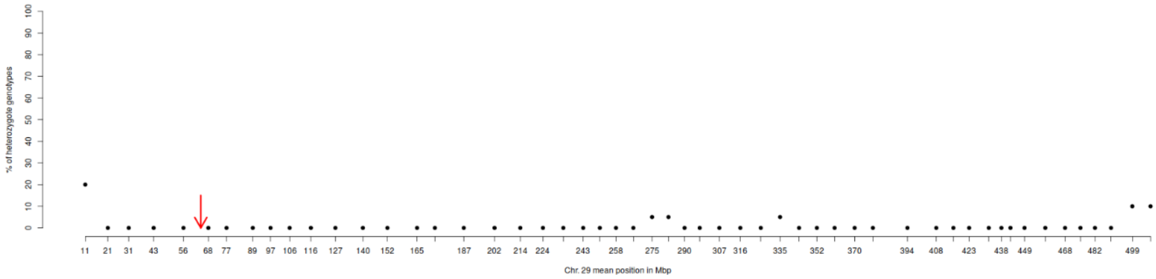

Embryo 3

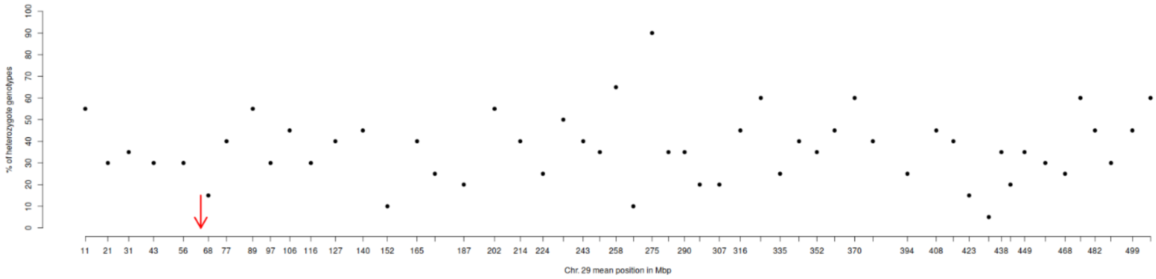

Embryo 4

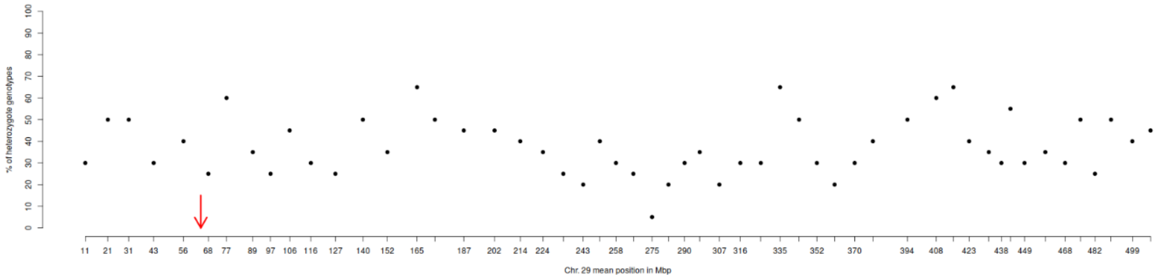

Embryo 5

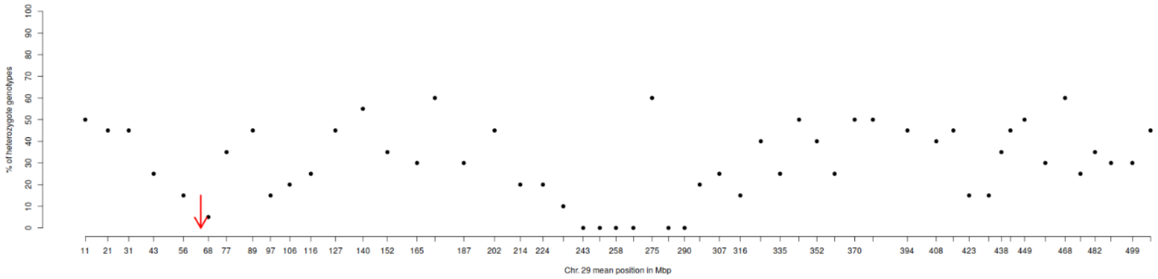

Embryo 6

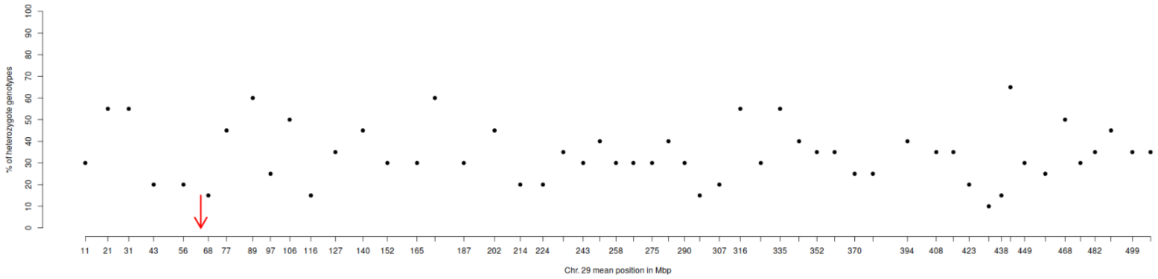

Embryo 7

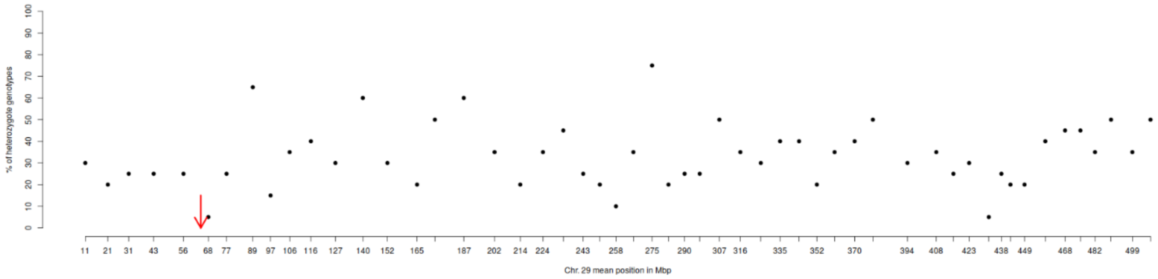

Embryo 8

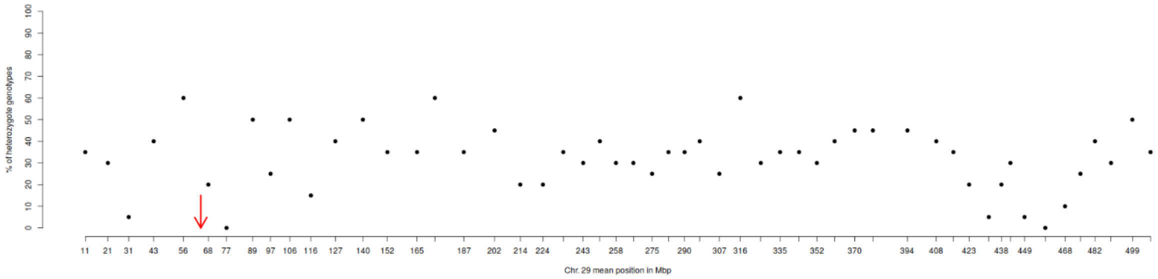

Embryo 9

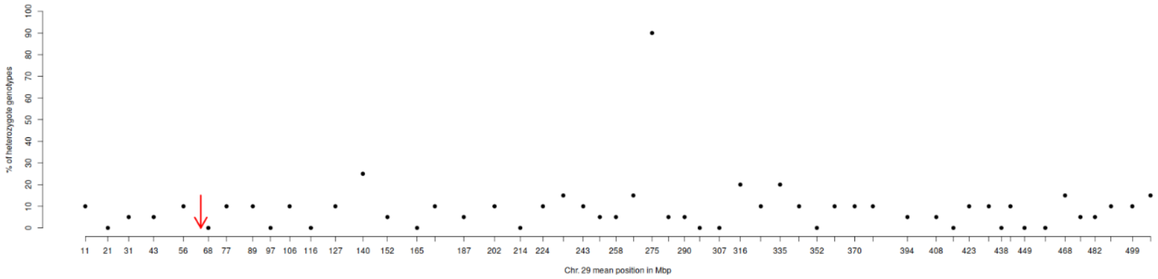

Embryo 10

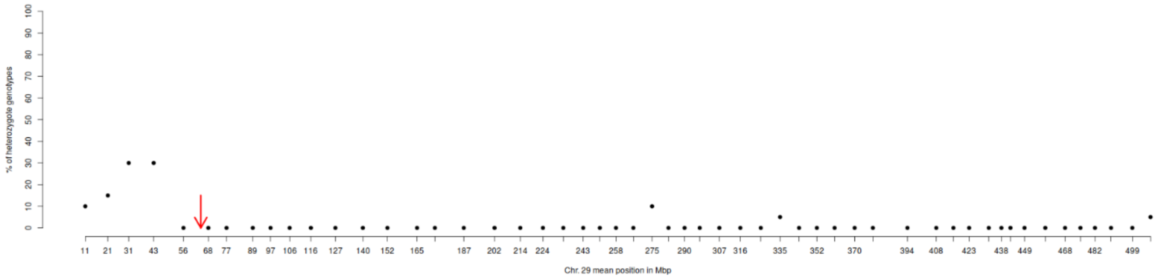

Embryo 11

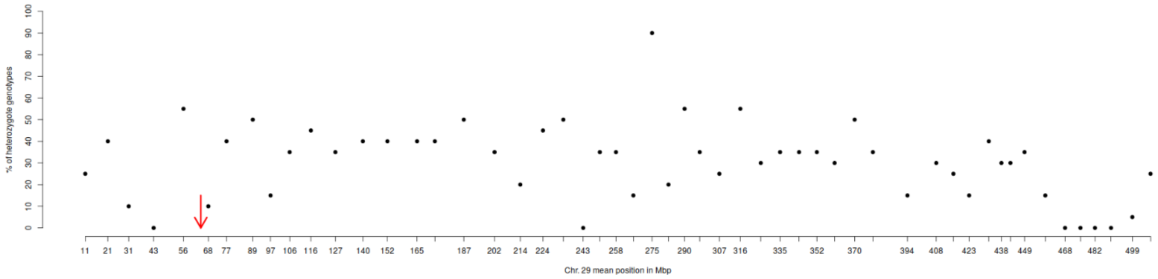

Embryo 12

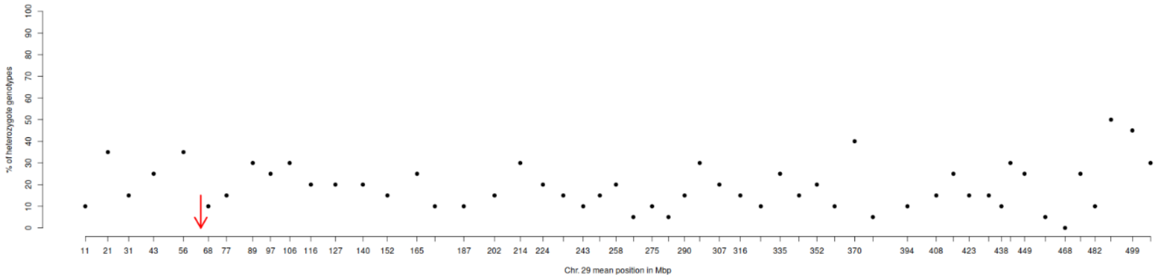

Embryo 13

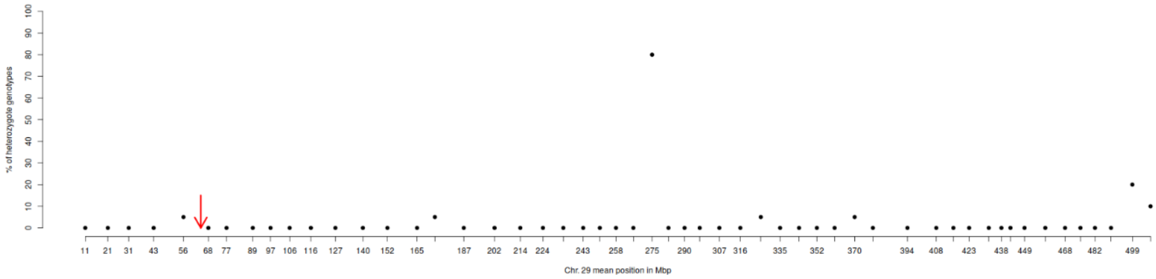

Embryo 14

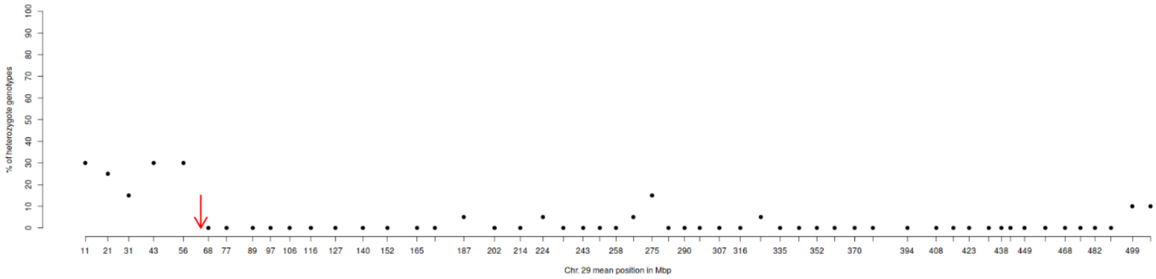

Embryo 15

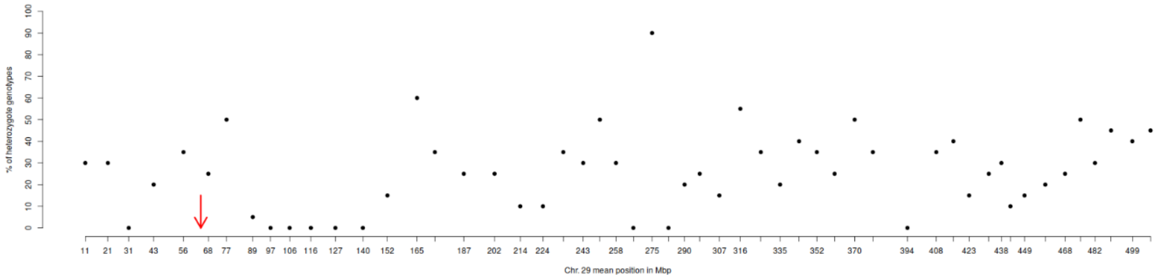

Embryo 16

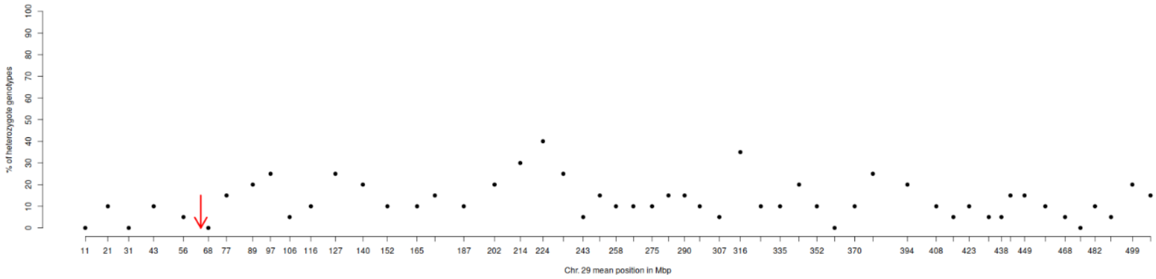

Embryo 17

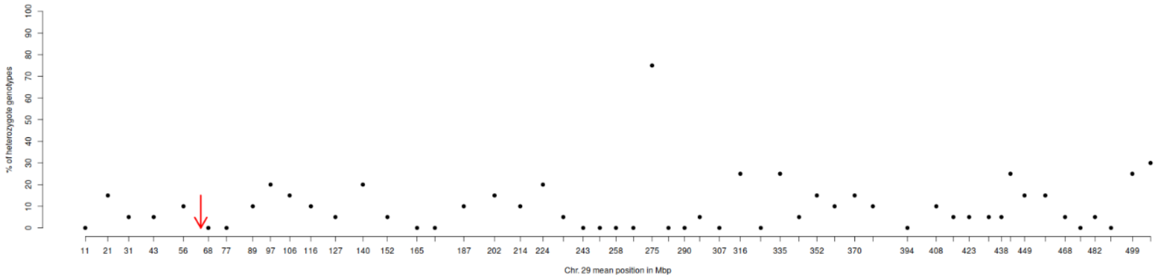

Embryo 18

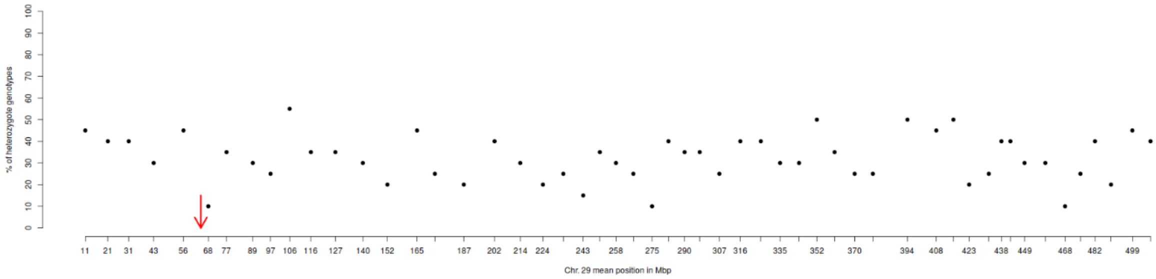

Embryo 19

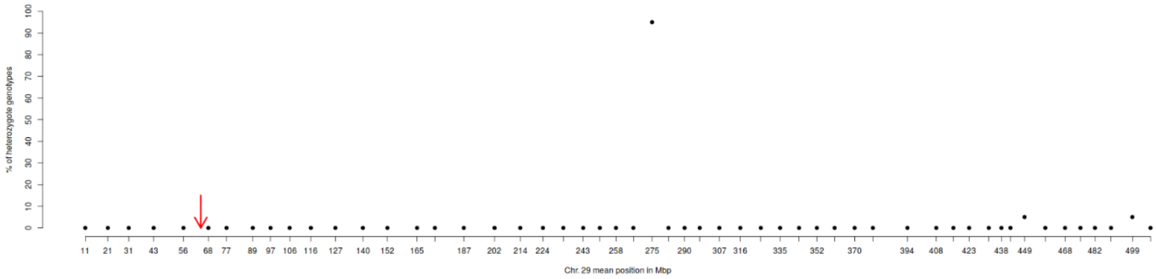

Embryo 20

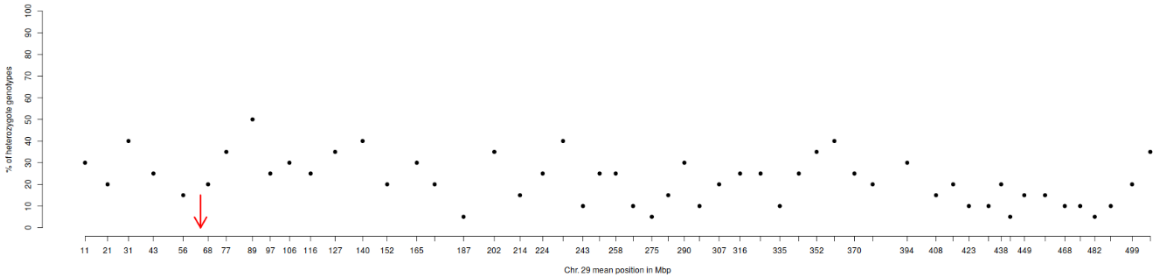

Embryo 21

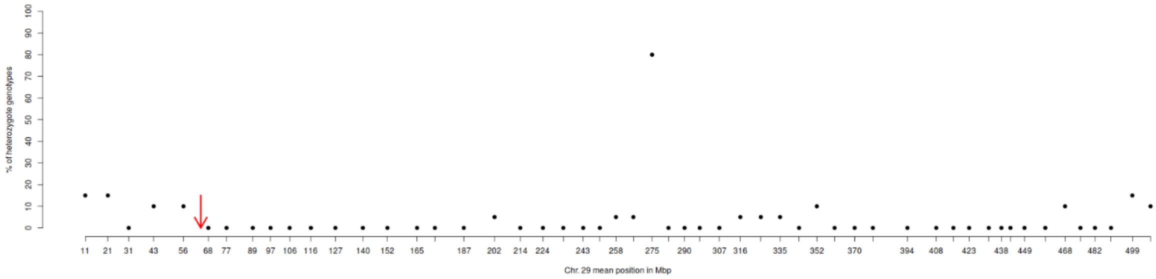

Embryo 22

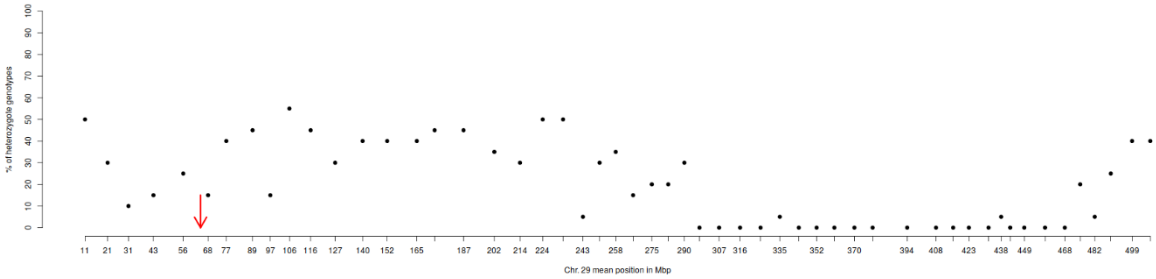

Embryo 23

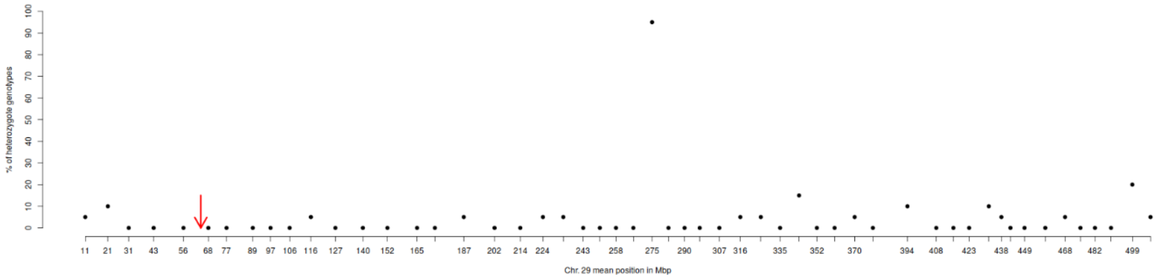

Supplement: Supplementary file 3 — Supplementary Figure 3. [file 41598_2022_14699_MOESM3_ESM.pdf]
